# Bosselated scalp nodules with alopecia

**DOI:** 10.1016/j.jdcr.2026.08.011

**Published:** 2026-08-17

**Authors:** Beatriz F. Vilela, Inês Aparício Martins, Joana Cabete

**Affiliations:** a CRI de Dermatovenereologia, Hospital de Santo António dos Capuchos, ULS São José, Lisbon, Portugal; b NOVA Medical School (NMS), Faculdade de Ciências Médicas (FCM), Universidade NOVA de Lisboa, Lisbon, Portugal

**Keywords:** Candida, deep fungal infection, eumycetoma, mycetoma, scalp

## Case description

A 27-year-old man, with no relevant medical history, presented with a 6-year history of progressively enlarging, painful scalp swellings associated with intermittent exudation. He reported gradual enlargement of the lesions over several years, with recurrent episodes of drainage. There were no systemic symptoms, including fever, weight loss, or night sweats.

On physical examination, multiple coalescent, soft, bosselated nodules were observed on the left parietal and occipital scalp, with overlying alopecia and focal serohemorrhagic crusts (Fig 1). The largest lesion measured approximately 12 cm in greatest diameter. The surrounding scalp showed areas of scarring alopecia. No cervical lymphadenopathy was detected. The remainder of the skin examination was unremarkable.

**Fig 1:**
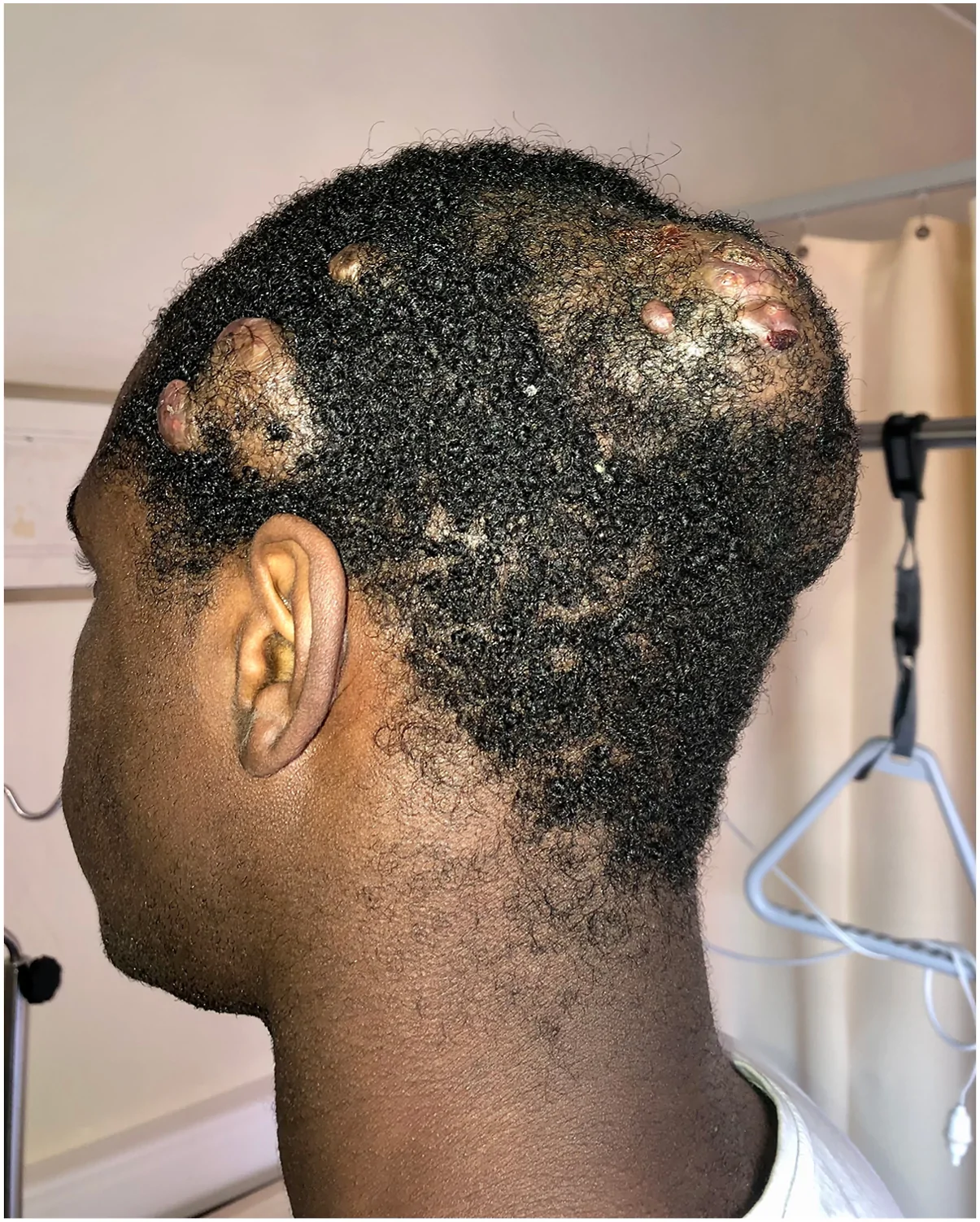
Multiple coalescent, bosselated nodules involving the left parietal and occipital scalp, with overlying alopecia and focal serohemorrhagic crusting.

Given the chronic course and the presence of large scalp nodules with intermittent drainage and alopecia, a deep infectious or inflammatory scalp disorder was suspected. A scalp biopsy was performed for histopathologic and microbiologic evaluation.

**Question: Which of the following is the most likely diagnosis?**

**A.** Dissecting cellulitis of the scalp
**B.** Kerion celsi
**C.** Mycetoma
**D.** Cutaneous tuberculosis
**E.** Botryomycosis

## Answer discussion

Correct answer: C. Mycetoma.

Histopathologic examination of the scalp biopsy (Fig 2) demonstrated fungal grains within deep suppurative granulomatous inflammation, confirming the diagnosis of mycetoma. Molecular analysis by polymerase chain reaction, performed on the biopsy specimen, identified *Candida zeylanoides* and *Candida lusitaniae*, supporting the diagnosis of eumycetoma. Prolonged oral itraconazole therapy was initiated, with clinical improvement; however, residual bosselated cicatricial lesions persisted after treatment.

**Fig 2:**
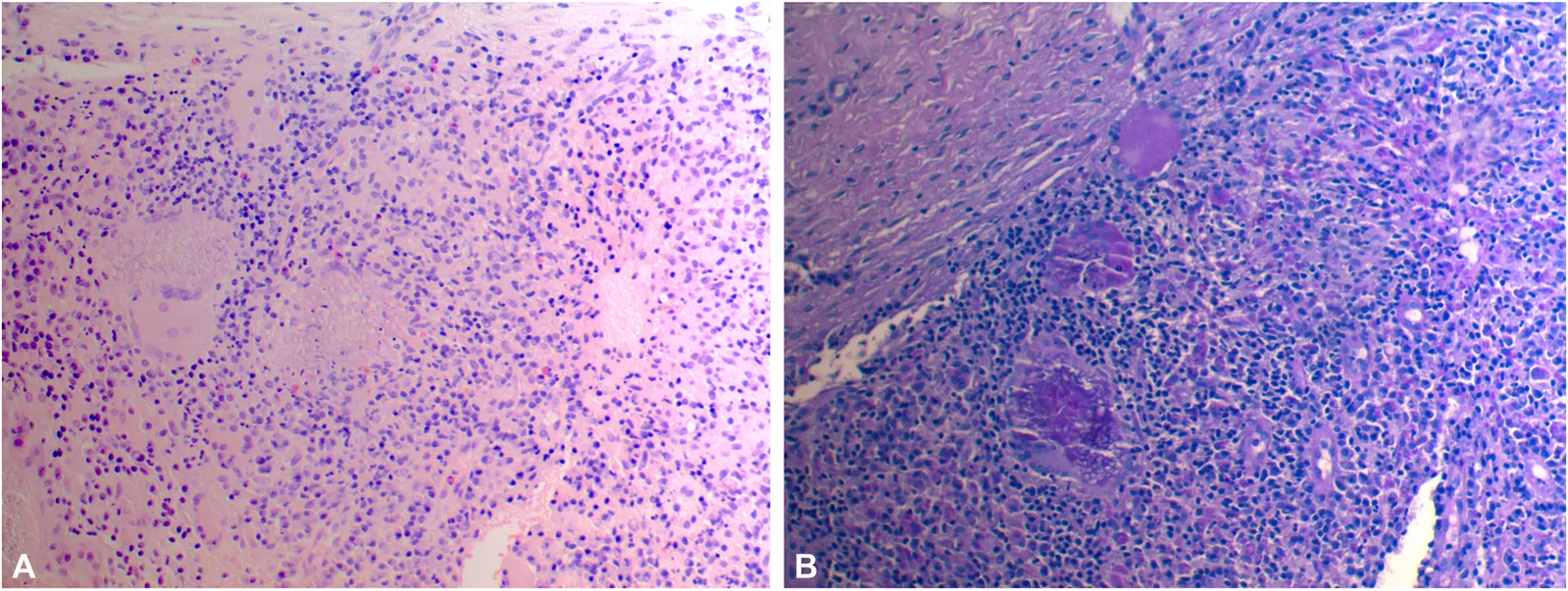
Histopathologic examination of the scalp biopsy. **A,** Deep dermal/subcutaneous suppurative granulomatous inflammation. **B,** Periodic acid–Schiff staining highlighting fungal grains/colonies, supporting the diagnosis of eumycetoma.

Mycetoma is a chronic, progressive infection of the skin and subcutaneous tissue caused by either filamentous bacteria, resulting in actinomycetoma, or fungi, resulting in eumycetoma. It classically presents with a triad of tumefaction, draining sinuses and grains. Although the feet and lower limbs are the most frequently affected sites, mycetoma may occur in unusual locations, including the scalp.^1^^,^^2^

This patient’s long-standing, progressively enlarging scalp nodules with intermittent suppurative drainage, bosselated morphology and overlying alopecia were important clinical clues. The identification of fungal organisms in biopsy tissue, rather than a superficial swab, supported their pathogenic role in this case.

The main differential diagnosis includes dissecting cellulitis of the scalp, kerion celsi, cutaneous tuberculosis, botryomycosis, and actinomycetoma. Dissecting cellulitis may present with painful nodules, abscesses, sinus tracts, and scarring alopecia, but usually occurs as part of the follicular occlusion spectrum and does not show fungal tissue invasion. Kerion celsi can cause inflammatory boggy plaques and alopecia, but its course is typically more acute or subacute. Cutaneous tuberculosis and atypical mycobacterial infections should be considered in chronic draining lesions, especially when ulceration or sinus tract formation is present. Botryomycosis may mimic mycetoma clinically, but is a chronic bacterial infection characterized by granules composed of bacteria rather than fungi. Clinically, actinomycetoma and eumycetoma may be very similar, both presenting with chronic swelling, draining sinuses, and grains. Therefore, clinical examination alone is usually insufficient to distinguish between them. Microbiologic and histopathologic confirmation is essential, because treatment differs substantially.^1^, ^3^, ^2^ Actinomycetoma is treated with prolonged antibacterial therapy, whereas eumycetoma usually requires prolonged systemic antifungal therapy. Surgical management may be considered depending on the extent of disease, response to medical therapy, and involvement of deeper structures.^1^^,^^3^

This case highlights the importance of considering mycetoma in chronic, suppurative, nodular scalp lesions with alopecia, even in nonclassical anatomic locations and in immunocompetent patients.

## Conflicts of interest

None disclosed.

## References

[1] Relhan V., Mahajan K., Agarwal P., Garg V.K. (2017). Mycetoma: an update. Indian J Dermatol.

[2] Zijlstra E.E., van de Sande W.W.J., Welsh O., Mahgoub E.S., Goodfellow M., Fahal A.H. (2016). Mycetoma: a unique neglected tropical disease. Lancet Infect Dis.

[3] Agarwal P., Jagati A., Rathod S.P., Kalra K., Patel S., Chaudhari M. (2021). Clinical features of mycetoma and the appropriate treatment options. Res Rep Trop Med.

